# High NEMO score values in nailfold videocapillaroscopy are associated with the subsequent development of ischaemic digital ulcers in patients with systemic sclerosis

**DOI:** 10.1186/s13075-020-02342-5

**Published:** 2020-10-13

**Authors:** Nicoletta Del Papa, Francesca Pignataro, Wanda Maglione, Antonina Minniti, Domenico Sambataro, Gianluca Sambataro, Gabriele Valentini, Roberto Caporali, Claudio Vitali

**Affiliations:** 1 Department of Rheumatology, UOC Day Hospital of Rheumatology, ASST G.Pini-CTO, 20122 Milan, Italy; 2 Department of Clinical and Experimental Medicine, Internal Medicine Unit, Section of Rheumatology, University of Catania, Catania, Italy; 3 Department of Clinical and Experimental Medicine, Regional Referral Center for Rare Lung Disease, University of Catania, Catania, Italy; 4 Department of Internal Medicine, Rheumatology Unit, 2nd University of Naples, Naples, Italy; 5 Department of Clinical Sciences and Community Health, Research Center for Adult and Pediatric Rheumatic Diseases, University of Milan, Milan, Italy; 6 Rheumatology Outpatient Clinics, ‘Mater Domini’ Humanitas Hospital, Castellanza, Italy

**Keywords:** Systemic sclerosis, Ischaemic digital ulcers, Nailfold videocapillaroscopy

## Abstract

**Background:**

Nailfold videocapillaroscopy (NVC) is a feasible method that allows the observation of the microvascular changes that mark the course of systemic sclerosis (SSc). In previous studies, we demonstrated that the NEMO score, i.e. the cumulative number of microhaemorrhages and microthromboses, is a good indicator of the steady-state level and overtime changes of disease activity (DA) in SSc.

**Objectives:**

To verify whether high NEMO scores, which mirror a very active microvascular derangement in the fingers, may be associated with the subsequent development of ischaemic digital ulcers (IDUs).

**Methods:**

The NEMO score was assessed at baseline (T0) in 98 patients with SSc, all classified according to the ACR-EULAR criteria. Of them, 90 were females, 48 had the limited and 50 had the diffuse cutaneous variant of SSc. Afterwards, the patients were closely followed up for 2 years, and the appearance of new IDUs recorded at any time of the follow-up. The T0-NEMO score values of patients who developed IDUs were compared to those of patients who did not. A receiver operating curve (ROC) was constructed, and the area under the curve (AUC) calculated by plotting the sensitivity and 1-specificity of the different NEMO score values in predicting the subsequent development of IDUs.

**Results:**

During the follow-up, 38 out of 98 patients developed one or more IDUs. The NEMO score at T0 was significantly higher in those who developed IDUs with respect to those who did not [median 14.5 (95% CI 11.0–21.5) and 4.5 (95% CI 4.0–6.0), respectively, *p* < 0.0001]. The ROC curve derived from different T0-NEMO score values had an AUC of 0.79 (95% CI 0.69–0.86, *p* < 0.0001). A NEMO score of ≥ 12 had a sensitivity of 83.3% (95% CI 71.5–91.7) and a specificity of 63.2% (95% CI 46.0–78.2), with positive (P) and negative (N) predictive (PV) values of 58.9% (95% CI 44.7–72.2) and 85.6% (71.8–94.4), respectively. A NEMO score of ≥ 16 had a sensitivity of 95.0% (95% CI 86.1–99.0) and a NPV of 93.4% (77.5–99.2).

**Conclusions:**

Being a valid tool to measure DA levels in SSc, the NEMO score also appears to be closely related to the subsequent development of IDUs in this disease.

## Introduction

Nailfold videocapillaroscopy (NVC) is a valid, feasible and non-invasive method to observe the abnormalities of the microvascular bed in different pathological conditions [1]. Specific features observed with this methodology have been found in some connective tissue diseases, namely in systemic sclerosis (SSc) [1–3]. Therefore, NVC has been considered a good tool to ascertain the diagnosis of SSc and has been included among the items composing the most recent classification criteria for this disease, defined by the American College of Rheumatology (ACR) and European League Against Rheumatism (EULAR) [4].

Furthermore, progressive changes of NVC features have been observed during the clinical course of SSc, and, as a consequence of this, different NVC patterns have been described in early, active and late phases of the disease [5]. According to these findings, the active phase of SSc is characterised by the prevalent presence of ectasic and giant capillaries, microhaemorrhages (MHEs) and microthromboses (MTs).

During the last few years, the NEMO score, i.e. the cumulative number of MHEs and MTs, has been proposed and validated as a good tool to assess both steady-state [6, 7] and overtime changes [8] of disease activity (DA) in the course of SSc. The NEMO score, in fact, showed a very close correlation with the composite scales which have been defined to assess this disease status entity, as those proposed by the European Scleroderma Study Group (EScSG) [9, 10] and, subsequently, by the European Scleroderma Clinical Trials and Research (EUSTAR) [11].

Since a more extensive involvement of microvascular bed in the active phase of SSc, which is correlated to the highest values of the NEMO score, may evolve to a more severe capillary loss and distal ischaemic damage, in the present study, we have investigated whether the highest levels of the NEMO score may be associated with the subsequent development of ischaemic digital ulcers (IDUs).

## Patients and methods

### Patients

The study was carried out in the cohort of patients with SSc that was used in the validation study of NEMO score. This cohort was initially composed of 102 patients with SSc who were referred to the Scleroderma Clinics of the Rheumatic Diseases Unit of the Gaetano Pini Institute of Milan. Since four of these patients dropped out from the follow-up, the present study was completed in 98 patients.

All enrolled patients met the ACR/EULAR classification criteria for SSc [4], and they were also sub-classified as having limited cutaneous SSc (lcSSc) or diffuse cutaneous SSc (dcSSc) according to the LeRoy et al. criteria [12]. At the time of the enrolment, it was preliminarily established to include around half the patients with inactive disease (EScSG score < 3) and a similar proportion of patients with active disease (EScSG score ≥ 3) [7].

The exclusion criteria were pre-existing conditions that may induce additional microvascular changes, such as diabetes, smoking and onychophagic habitus, presence of anti-phospholipid antibodies and pregnancy [13–16]. Another exclusion criterion was current treatment with beta-blockers which may exacerbate Raynaud’s phenomenon (RP) [17].

At the time of the study enrolment, all of the patients were receiving low-dose acetylsalicylic acid and calcium channel blockers (CCBs), and in addition, around one third of the patients were on treatment with other vasoactive agents (31 with a monthly infusion of iloprost, 5 with a weekly infusion of alprostadil, 7 with oral bosentan and 4 with sildenafil). None of the patients was taking anticoagulant therapy. Three out of these latter patients had pulmonary hypertension. For ethical reasons, these vasoactive therapies were maintained throughout the study.

### Nailfold videocapillaroscopy

NVC was performed in all the patients at enrolment time (T0) by using a videocapillaroscope with a × 200 magnification lens. All fingers of both hands, excluding thumbs, of each patient were examined by positioning each digit in such a way that the capillaroscopic light was 90° incident on the centre of the nailfold. Four adjoining 1-mm fields—two on the right and two on the left side, starting from the middle of the nailfold and for a total extension of 4 mm—were examined [6, 7]. The derived digital images were stored using dedicated software (VideoCap; Scalar Co. Ltd., Tokyo, Japan). One experienced investigator (FP) was responsible for reviewing and scoring the stored NVC images of all the study patients, according to the NEMO definition [8].

### Assessment of digital ulcers

All the enrolled patients were carefully observed every 3 months for the following 2 years, with particular attention given to the new appearance of at least one IDU in the distal fingers. An IDU was defined as a painful area, of at least 6 mm in diameter at its longest point, with visible depth and loss of dermis, located at the volar surface of the digit, distal to the proximal interphalangeal digital crease.

### Statistical analysis

Statistical analysis was performed using MedCalc software package, 2014 version (MedCalc® Inc., Ostend, Belgium).

The Mann-Whitney test was applied to compare T0-NEMO score values recorded in patients who subsequently developed IDUs to those assessed in patients who did not. The T0-NEMO score values were also analysed in sub-groups of patients subdivided according to their NVC patterns and SSc cutaneous variants. This non-parametric method was adopted because the NEMO score variable did not have a normal distribution (Shapiro-Wilk test, *p* > 0.05).

Other variables such as disease duration, NVC patterns, SSc cutaneous variants, autoantibody specificities, history of previous ulcers and types of vasoactive therapies were also tested for a possible association with the subsequent development of IDUs, using the chi-square crosstabs for categorical variables and the Mann-Whitney test for discrete variables.

Receiver operating characteristic (ROC) curves were constructed by plotting the sensitivity and 1-specificity values of the T0-NEMO scores in identifying patients who developed IDUs during the following 2-year observation time. The area under the curve (AUC) was calculated together with the related 95% confidence intervals (CI) by applying the Hanley-McNeil test.

We also identified the sensitivity, specificity and positive (P) and negative (N) predictive values (PV) of the different T0-NEMO scores with the best performance in capturing patients who developed IDUs in the subsequent follow-up observation.

The Kaplan-Meier curve analysis and log rank test were used to evaluate the occurrence and the time of the appearance of IDUs during the follow-up in patients having different levels of T0-NEMO score. Hazard ratio (HR) with the related 95% CI was calculated and used for the comparison between the two curves.

Finally, a logistic regression model was tested in which the appearance of new digital ulcers in the follow-up represented the independent variable, whilst dependent variables were those that showed to be separately associated with the development of IDUs.

## Results

The clinical characteristics of the patients included in this study are reported in Table 1.

**Table 1:** Demographic and clinical characteristics of the cohort of patients with SSc enrolled in the study

**Numbers of patients**	98
**Male/female**	8/90
**Median age, years (range)**	58 (21–84)
**Median disease duration, years (range)**	6 (0–26)
**lcSSc/dcSSc**	48/50
**Autoantibodies**	
**ACA,** ***n*** **(%)**	42 (42.8)
**Anti-topoisomerase I,** ***n*** **(%)**	50 (51)
**Others,** ***n*** **(%)**	6 (6.1)
**NVC patterns**	
**Early,** ***n*** **(%)**	16 (16.2)
**Active,** ***n*** **(%)**	42 (42.8)
**Late,** ***n*** **(%)**	40 (41)
**Patients on prostanoid therapy,** ***n*** **(%)**	36 (36.7)
**Patients on bosentan/sildenafil therapy,** ***n*** **(%)**	11 (11.2)

*dcSSc* diffuse cutaneous systemic sclerosis, *lcSSc* limited cutaneous systemic sclerosis, *ACA* anti-centromere antibody, *NVC* nailfold videocapillaroscopy

The cohort was composed of 98 patients classified as having SSc according to the ACR/EULAR criteria [4]. They were aged between 21 and 84 years, with a large prevalence of females (ratio 10/1) and a comparable number of patients having lcSSc and dcSSc (48 and 50, respectively). The NVC pattern [5] was defined as early, active and late in 16, 42 and 40 patients, respectively. Twenty four out of 98 patients experienced one or more IDUs before their enrolment in the study. Anti-centromere and anti-topoisomerase I antibodies were positive in 42 and 50 patients, respectively.

**Fig. 1:**
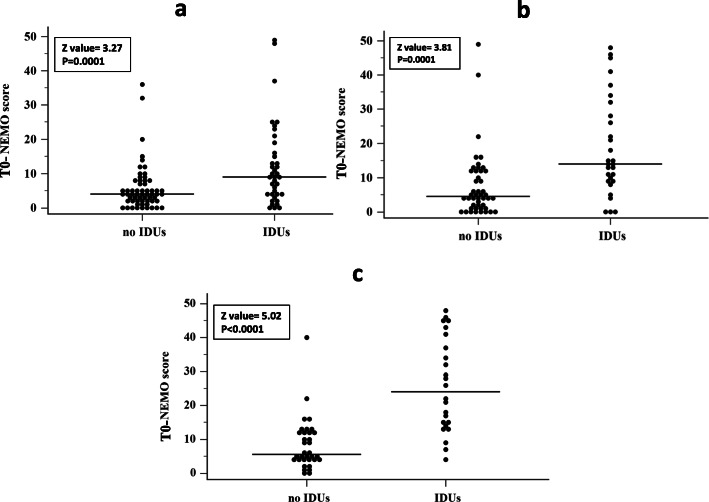
Distribution plots of NEMO score values in patients with SSc who developed IDUs and in those who did not (no IDUs) in the subsequent 2-year follow-up. The results obtained in the totality of patients (98 cases), in those naive for the occurrence of digital ulcers before enrolment (74 cases) and in those with early and active NVC patterns (58 cases) at the baseline are graphed in the left (**a**), right (**b**) and lower (**c**) parts of the figure, respectively. Horizontal lines represent the median values. Significance of the differences is also reported (*p* > 0001 in all cases), by the Mann-Whitney test. For abbreviations, see text

During the follow-up, 38 out of 98 patients (38.8%) developed one or more new IDUs. The T0-NEMO scores were significantly more elevated in the patients who developed IDUs with respect to those who did not [median 14.5 (95% CI 11.0–21.5) and 4.5 (95% CI 4.0–6.0), respectively, *p* < 0.0001] (Fig. 1a). As expected, the T0-NEMO scores were significantly higher in patients with NVC active pattern [median 13.0 (95% CI 10.4–16.8)] with respect to those with early NVC pattern [median 5.5 (95% CI 1.6–12.4), *p* < 0.02] and late NVC pattern [median 4.0 (95% CI 1.0–6.0), *p* < 0.0001]. No difference in the T0-NEMO score values was recorded between patients with early and late NVC patterns (*p* = 0.23). Finally, the T0-NEMO scores were significantly more elevated in patients with dcSSc in comparison with patients with lcSSc [median 12.0 (95% CI 9.0–15.0) vs 5.0 (95% CI 4.0–6.0), *p* < 0.003].

The appearance of new IDUs was associated with the history of previous ulcers and with the presence of the diffuse cutaneous variant of the disorder (chi-square 15.6, *p* < 0.0001, and 7.8, *p* < 0.01, respectively). A similar association was found with the presence of anti-topoisomerasi I antibodies that are notoriously closely related with the dcSSc variant of the disorder. Conversely, no association was found between the appearance of IDUs and disease duration or use of more aggressive vasoactive therapies (monthly iloprost infusion, oral bosentan or sildenafil) in addition to basal CCBs. IDU development during the follow-up was observed in 4/16 (25.0%), 20/42 (47.6%) and 14/40 (35.0%) patients with early, active and late NVC pattern, respectively. No statistically significant difference in the IDU prevalence between these NVC groups was recorded.

A logistic regression model in which all the three variables independently linked with subsequent appearance of IDUs were taken into account (T0-NEMO score, history of previous IDUs and dcSSc variant) showed that only the history of previous ulcers and T0-NEMO gave a significant contribution to the model, whilst the third variable was discharged (see Table 2).

**Table 2:** Logistic regression model in which the subsequent appearance of IDUs was the dependent variable and the NEMO score values, history of previous IDUs and the presence of dcSSc variant represented the independent variables

Total number of patients with SSc	98	
Number of patients who developed new IDUs	38	
Number of patients who did not develop a new IDU	60	
Independent variables	Coefficient (standard error)	*p*
T0-NEMO score	0.12 (0.03)	= 0.0001
Previous IDUs	2.63 (0.63)	< 0.0001
dcSSc variant	discharged	–
Constant	− 2.52	
Full model likelihood	Chi-square	*p*
98.32	44.56	< 0.0001

**Fig. 2:**
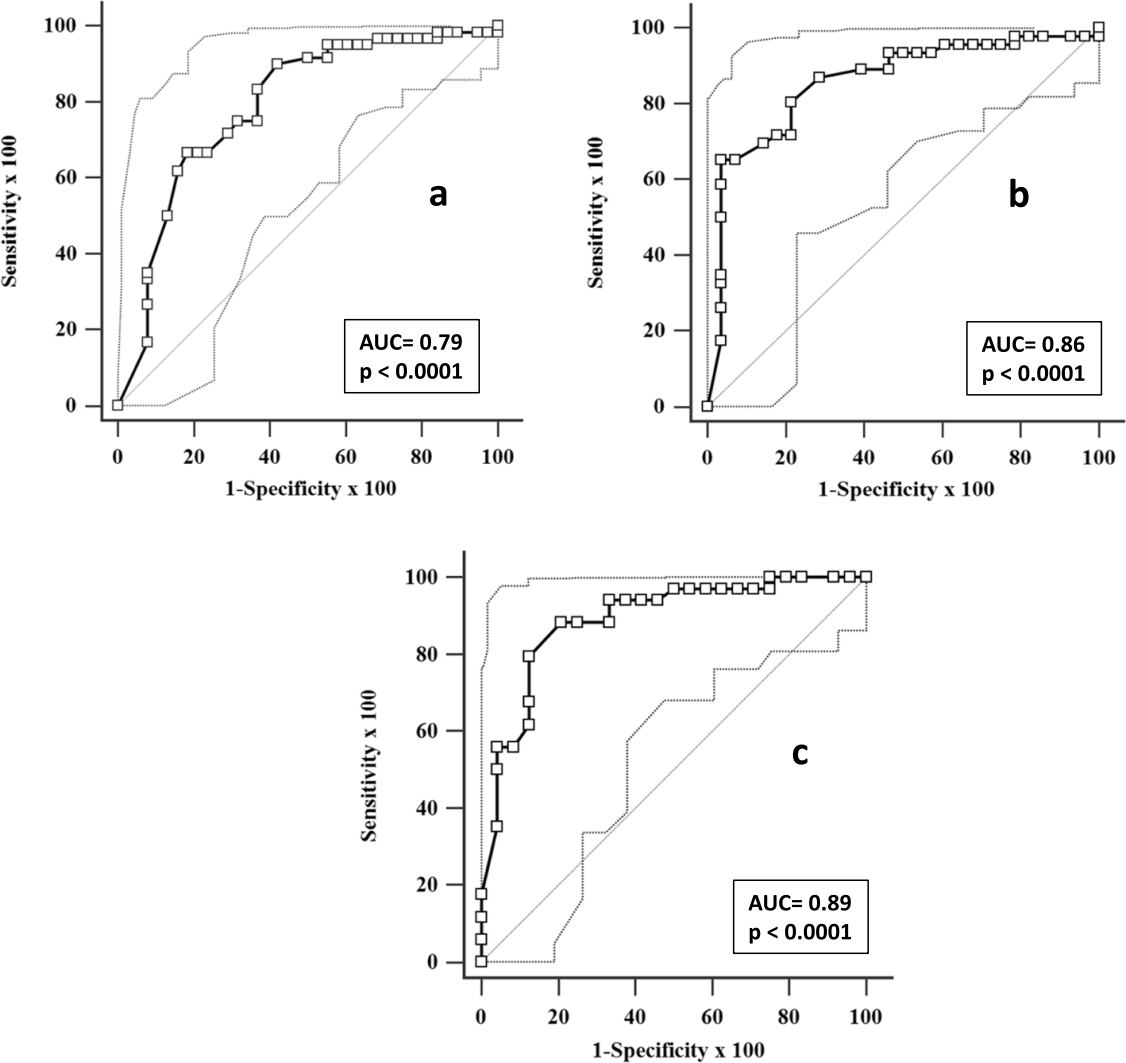
ROC curve analysis of sensitivity and 1-specificity values of the T0-NEMO score in predicting the development of IDUs during the subsequent 2-year-follow-up. In the upper left (**a**), and right (**b**) parts of the figure, the ROC curves obtained in the totality of patients and in those naive for the occurrence of IDUs before the study enrolment are separately graphed. The ROC curve obtained analysing only patients with early and active NVC patterns is graphed in the lower part (**c**) of the figure. Dotted lines represent the 95% CI of the curves. The corresponding AUC values of the ROC curves are also reported. For abbreviations, see text

The ROC curve obtained by plotting sensitivity and 1-specificity of the different T0-NEMO score values in identifying patients who developed IDUs is represented in Fig. 2a. The AUC of this ROC curve was 0.79 (95% CI 0.69–0.86, *p* < 0.0001).

A NEMO score of 12 or more showed a sensitivity of 83.3% (95% CI 71.5–91.7) and a specificity of 63.2% (46.0–78.2), with a PPV and NPV of 58.9% (95% CI 44.7–72.2) and 85.6% (71.8–94.4), respectively. Furthermore, a NEMO score of 16 or more was highly predictive of future development of IDUs showing, in this respect, a sensitivity of 95.0% (95% CI 86.1–99.0) and a NPV of 93.4% (95% CI 77.5–99.2).

**Fig. 3:**
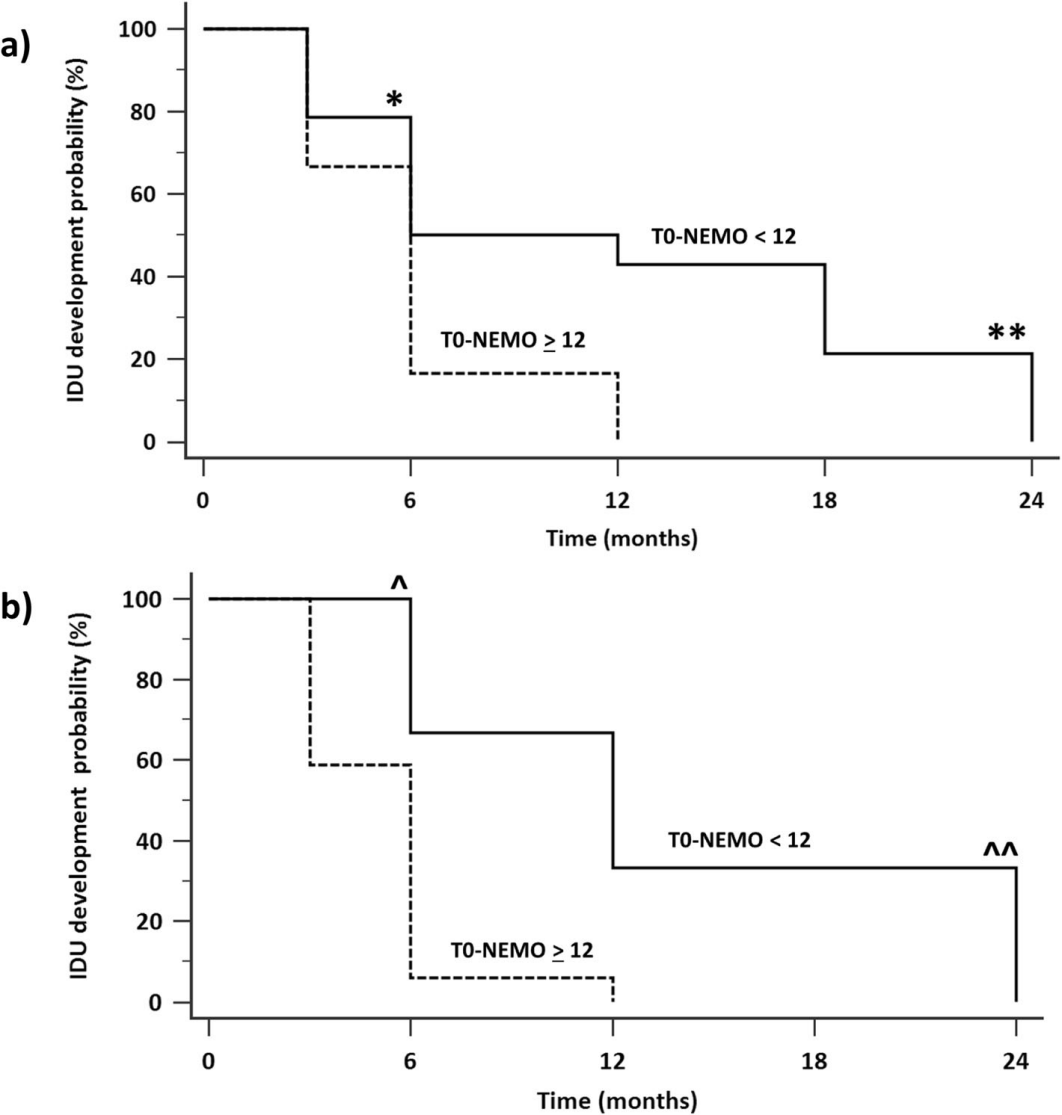
**a** Progressive occurrence of IDUs in patients with a T0-NEMO score of ≥ 12 (lower curve) and < 12 (upper curve) is analysed by the Kaplan-Meier survival analysis and log rank test. The risk of developing new IDUs is significantly higher in patients with a T0-NEMO score of ≥ 12 just at 6 months (*) [HR 1.42 (95% CI 0.71–2.86), *p* = 0.03] and at 24 months (**) [HR 1.86 (95% CI 0.98–3.5), *p* = 0.005]. **b**. The Kaplan-Meyer survival analysis performed in patients without previous IDUs gave similar results. Even in this group of patients, the risk for developing new IDUs was significantly higher in those with a T0-NEMO score of ≥ 12 both at 6 months (^) [HR 2.26 (95% CI 0.42–12.2), *p* < 0.01] and at 24 months (^^) [HR 2.25 (95% CI 0.85–5.96), *p* < 0.02]

The Kaplan-Meier curve analysis confirmed that the development of subsequent IDUs was significantly more frequent in patients having a T0-NEMO score of 12 or more with respect to those with a lower baseline NEMO score. The difference between the two curves became significant after 6 months from T0 (Fig. 3a).

When the same statistical analysis was limited to the 74 patients who had no previous occurrence of IDUs, the results were very similar. Twenty-eight of them (37.8%) developed at least one IDU during the 2-year follow-up. The T0-NEMO scores had a median value of 14.0 (95% CI 10.8–22.9) in the patients who developed IDUs and of 4.5 (95% CI 3.9–6.3) in those who did not (*p* < 0.0001) (Fig. 1b). The AUC of the ROC curve (Fig. 2b) was slightly higher [0.86 (95% CI 0.76–0.93), *p* < 0.0001] than that found taking into consideration the totality of patients. This difference, however, was not significant (*z* value 1.01, *p* = 0.3). In this more restricted cohort, a NEMO score of 12 or more had a sensitivity of 80.4% (95% CI 66.1–90.6) and a specificity of 78.6% (95% CI 59.0–91.7), whilst a NEMO score of 16 or more maintained a strong predictive value of future development of IDUs showing a sensitivity of 93.5% (95% CI 82.1–98.6) and a NPV of 93.1% (95% CI 76.2–99.3).

A Kaplan-Meyer curve was also built and analysed in patients who were naive for previous IDUs. Even in this case, the appearance of IDUs was more frequent in patients having a T0-NEMO score of ≥ 12, and this difference was comparably significant at 6 and 24 months (Fig. 3b).

The statistical analysis was also performed in patients with early and active NVC pattern, thus excluding those patients with late NVC pattern who had more pronounced fibrotic changes.

In this selected cohort, the T0-NEMO scores had a median value of 24.0 (95% CI 15.0–34.5) in patients who developed IDUs and of 5.5 (95% CI 4.0–10.3) in those who did not (*p* < 0.0001) (Fig. 1c). The AUC of the ROC curve (Fig. 2c) was again slightly higher [0.89 (95% CI 0.78–0.96), but not significantly different from that obtained in the whole group of patients (*z* value 1.45, *p* = 0.14). A NEMO score of 16 or more was again very strongly associated with future development of IDUs, showing a sensitivity of 94.1 (95% CI 82.4–97.4) and a NPV of 94.1 (95% CI 76.4–99.6).

## Discussion

In previous studies, we demonstrated that the NEMO score, computed during NVC examination, is a good tool to measure both steady-state and overtime changes of DA in SSc [6–8], being strictly correlated with the multi-item scales proposed to measure the same disease status entity [9–11].

In this study, we demonstrate that high values of the NEMO score are also associated with the future development of IDUs in patients with SSc. Patients with a NEMO score of 16 or more have a probability of incidental IDUs of around 95% in the subsequent 2 years.

When the ROC curve analysis was carried out, the performance of the NEMO score as an indicator of future development of IDUs is slightly higher, although not significantly different, when the analysis was limited to patients who were naive for the previous occurrence of IDUs and to those with early and active NVC patterns. The present results indicate that high levels of NEMO score are able to forecast the future development of IDUs independently of the fact that the patients experienced or not previous ulcers. However, the present data confirm that the history of previous ulcers is associated with the following development of new ulcers.

In a previous study, Sebastiani et al. [18] demonstrated that the development of IDUs in the following 3 months was associated with a low number of capillaries and a high number of megacapillaries in NVC. These data are not contradictory with respect to the present ones. It is plausible that a late pattern of NVC is able to forecast the development of IDUs in the subsequent short time, whilst high NEMO score values are related with a later appearance of IDUs as shown by the survival curve in the present study (Fig. 3). This statement is supported by the present knowledge on the evolution of microvascular lesions in SSc. Endothelial damage is considered the first step of the evolving pathological process in this disease [19–21], and in the later phase, the destiny of many capillaries is thrombotic obliteration followed by extravasation. Therefore, multiple MTs and MHEs which are observed in NVC, aligned distally in the cuticle, can be the mirror of the synchronous pathological aggression of many capillaries [22]. In this view, the NEMO score can be considered as a relatively simple method to quantify this phenomenon.

The subsequent response to the initial microvascular damage in SSc is the loss of capillaries. The compensatory dilatation of residual capillary loops with the formation of enlarged capillaries and GCs is believed as an ineffective tentative process of capillary regeneration [23]. The final step of the process is a sort of capillary desertification accompanied by fibrotic changes. On the basis of this sequence of pathological events, one can presume that the higher the NEMO score values, the greater the possibility that the following fibrotic and ischaemic changes will be more severe and rapid. Thus, it is not surprising that the highest NEMO score values can anticipate the subsequent development of IDUs.

The occurrence of IDUs on the fingertips which has been observed in around half of the patients with SSc [24–26] commonly leads to a significant worsening of the patient’s quality of life often causing often severe pain and difficulties in performing the simplest daily living activities. The healing of IDUs is frequently a lengthy process, requiring accurate and intensive topical and systemic treatment [25].

Therapeutic measures, aimed at avoiding or limiting the development of IDUs in patients with SSc, usually consist of the use of vasoactive agents such as CCBs, prostanoids, endothelin receptor antagonists and phosphodiesterasis-5 inhibitors. For all these agents, there is no evidence of effectiveness in the primary prevention of IDU development, whilst several studies have demonstrated their beneficial effects in preventing new IDUs and in favouring the healing of active ulcers [27, 28].

The present study shows that slightly less than 40% of the patients who were naive for previous IDUs developed this kind of lesions in the follow-up, and in a significant proportion of them, this happens after few months. One can postulate that a more aggressive vasoactive therapy could be reserved to patients with very high NEMO score values (for instance, those with 16 or more NEMO score values at baseline). These patients have around 95% of probability of future occurrence of IDUs, even in a relatively short time.

This is a retrospective study in a relatively limited cohort of patients collected in a unique centre. The results of this study need to be confirmed in a prospective multicentre study that should include a larger cohort of SSc patients.

## Conclusions

The NEMO score, which has been previously proposed as a valid tool to measure DA in SSc, demonstrated that it is also able to identify patients who, having the highest values of this NVC index, are candidates for the subsequent development of IDUs. The identification of this subset of patients suggests that more aggressive therapies could be reserved to them to prevent the subsequent appearance of such a painful and life-threatening complication.

## Data Availability

The datasets used and analyses made during the current study are available from the corresponding author on reasonable request.

## Abbreviations

ACA: Anti-centromere antibodies
ACR/EULAR: American College of Rheumatology/European League Against Rheumatism
AUC: Area under the curve
CCBs: Calcium channel blockers
CI: Confidence intervals
DA: Disease activity
dcSSc: Diffuse cutaneous systemic sclerosis
EScSG: European Scleroderma Study Group
EUSTAR: European Scleroderma Trial and Research
GCs: Giant capillaries
IDUs: Ischaemic digital ulcers
lcSSc: Limited cutaneous systemic sclerosis
MHEs: Microhaemorrhages
MTs: Microthrombosis
NEMO: Number of microhaemorrhages and microthromboses
NPV: Negative predictive values
NVC: Nailfold videocapillaroscopy
PPV: Positive predictive values
ROC: Receiver operating characteristic
SSc: Systemic sclerosis
